# PPARβ/δ and PPARγ maintain undifferentiated phenotypes of mouse adult neural precursor cells from the subventricular zone

**DOI:** 10.3389/fncel.2015.00078

**Published:** 2015-03-18

**Authors:** Carolina Bernal, Claudia Araya, Verónica Palma, Miguel Bronfman

**Affiliations:** ^1^Department of Cell and Molecular Biology, Faculty of Biological Sciences, Center for Aging and Regeneration, Pontifical Catholic University of ChileSantiago, Chile; ^2^Laboratory of Stem Cells and Development, Faculty of Science, FONDAP Center for Genome Regulation, University of ChileSantiago, Chile

**Keywords:** neural stem/precursor cells, PPARβ/δ, PPARγ, subventricular zone, SOX2, EGFR

## Abstract

The subventricular zone (SVZ) is one of the main niches of neural stem cells in the adult mammalian brain. Stem and precursor cells in this region are the source for neurogenesis and oligodendrogesis, mainly in the olfactory bulb and corpus callosum, respectively. The identification of the molecular components regulating the decision of these cells to differentiate or maintain an undifferentiated state is important in order to understand the modulation of neurogenic processes in physiological and pathological conditions. PPARs are a group of transcription factors, activated by lipid ligands, with important functions in cellular differentiation and proliferation in several tissues. In this work, we demonstrate that mouse adult neural precursor cells (NPCs), *in situ* and *in vitro*, express PPARβ/δ and PPARγ. Pharmacological activation of both PPARs isoforms induces proliferation and maintenance of the undifferentiated phenotype. Congruently, inhibition of PPARβ/δ and PPARγ results in a decrease of proliferation and loss of the undifferentiated phenotype. Interestingly, PPARγ regulates the level of EGFR in adult NPCs, concurrent with it is function described in embryonic NPCs. Furthermore, we describe for the first time that PPARβ/δ regulates SOX2 level in adult NPCs, probably through a direct transcriptional regulation, as we identified two putative PPAR response elements in the promoter region of Sox2. EGFR and SOX2 are key players in neural stem/precursor cells self-renewal. Finally, rosiglitazone, a PPARγ ligand, increases PPARβ/δ level, suggesting a possible cooperation between these two PPARs in the control of cell fate behavior. Our work contributes to the understanding of the molecular mechanisms associated to neural cell fate decision and places PPARβ/δ and PPARγ as interesting new targets of modulation of mammalian brain homeostasis.

## Introduction

The subventricular zone of the lateral ventricles (SVZ) in the adult mammalian brain is one of two major CNS neural stem cell niches. There, neural stem cells self-renew and differentiate into neurons, oligodendrocytes and astrocytes (Gage et al., 1998; Doetsch et al., 1999; Rietze et al., 2001; Rietze and Reynolds, 2006). Within the SVZ, Epidermal Growth Factor Receptor (EGFR) expressing stem cells (type B cells) give rise to rapidly dividing transit-amplifying progeny (type C cells) which also express EGFR, which in turn generate immature neuroblasts (type A cells) (Doetsch et al., 2002). B and C cell populations are responsible of making neurospheres in cultures (Doetsch et al., 2002; Pastrana et al., 2009); and will be referred here as neural precursor cells (NPCs). Regulation of NPCs cell fate involves a complex and coordinated network of extrinsic and intrinsic signaling pathways. Among the molecules that regulate NPCs pool proliferation and maintenance, Sonic Hedgehog (Shh) and Epidermal Growth Factor (EGF) have been addressed as important mitogenic signals (Reynolds et al., 1992; Doetsch et al., 2002; Palma et al., 2005; Reinchisi et al., 2013; Alvarez-Buylla and Ihrie, 2014). In NPCs, EGFR signaling functions as a control system of cell proliferation, not only by responding to its own ligands but also by serving as a nodal element for a variety of other stimuli acting through downstream effectors of EGFR signaling pathways (Sibilia et al., 2007; Hu et al., 2010; Reinchisi et al., 2013). Cell-intrinsic components committed in cell fate decisions involve transcription factors, some of which play a role in self-renewal, such as SOX2, BMI1 and TLX (Graham et al., 2003; Ferri et al., 2004; Shi et al., 2004; Molofsky et al., 2005; Qu et al., 2010), while others, like MASH1 (Casarosa et al., 1999; Cau et al., 2002), act in neuronal differentiation. Although much progress has been made in understanding NPCs function in the adult brain, the detailed understanding of events regulating the delicate balance between self-renewal capacity and stem cell fate is still far from being clarified.

Peroxisome proliferator-activated receptors (PPARs), a subgroup of the nuclear receptor superfamily, are ligand-activated transcription factors (Issemann and Green, 1990, 1991). Three isotypes of PPARs have been described in vertebrates, PPARα, PPARβ/δ and PPARγ, which are highly conserved between species (Issemann and Green, 1990, 1991; Dreyer et al., 1992; Schmidt et al., 1992; Kliewer et al., 1994). PPARα is barely expressed in the central nervous system of adult rodents (Braissant et al., 1996). In contrast, a high expression level of PPARβ/δ is observed in the developing neural tube and epidermis Braissant and Wahli, 1998; Keller et al., 2000). In adult rodents, PPARβ/δ is abundantly and ubiquitously expressed, although some tissues such as brain, adipose tissue, and skin have higher mRNA level (Kliewer et al., 1994; Braissant et al., 1996). PPARβ/δ has important functions in proliferation, differentiation and cellular survival in several cell types. In skin, induction of keratinocyte proliferation by several stimuli, such as tetradecanoylphorbol acetate, is associated with up-regulation of PPARβ/δ level in the epidermis (Michalik et al., 2005). Moreover, PPARβ/δ-mutants decrease the number of proliferative keratinocytes and display increased apoptosis in early hair follicles (Di-Poi et al., 2005). PPARβ/δ-null mice are also smaller than wild type littermates and their brains present alterations in the myelinization of the corpus callosum (Peters et al., 2000). Although PPARβ/δ is abundantly expressed in the brain, a possible role of this factor modulating NPCs behavior has not yet been studied.

Regarding PPARγ, expression pattern analysis of this factor shows a transient peak of expression in the central nervous system between E13.5 and E15.5 (Braissant and Wahli, 1998; Keller et al., 2000). Interestingly, NPCs cultures (neurosphere assay) obtained at E13.5 from PPARγ +/− mice shows diminished cellular viability and EGFR level (Wada et al., 2006). On the other hand, NPCs obtained from wild type embryos and treated with rosiglitazone (PPARγ agonist) increase cellular viability and EGFR level, suggesting a role of this isotype in NPCs self-renewal (Wada et al., 2006). *In vivo* treatments with PPARγ agonists, namely pioglitazone and rosiglitazone, also increase both cellular proliferation and differentiation in the SVZ (Morales-Garcia et al., 2011). Recently, Ghoochani et al. evaluated PPARγ level during induced neuronal differentiation of mouse embryonic stem cells (mESC) *in vitro*. They observed an increase in PPARγ level in NPCs, which dropped in mature neurons. PPARγ antagonist decreased the expression of terminal differentiation markers suggesting a role of this transcription factor in the maintenance of the neural stem/precursor phenotype (Ghoochani et al., 2012).

The aim of the present study was to evaluate the potential role of PPARβ/δ and PPARγ in mouse adult NPCs. Our experiments establish the presence of both receptors in precursor cells *in situ* and *in vitro*. We also show that PPARγ regulates proliferation and maintenance of the precursor phenotype and modulates EGFR level in adult NPCs, a result that complements the function of this factor in embryonic mice NPCs (Wada et al., 2006). Finally, we describe for the first time that PPARβ/δ maintains the undifferentiated phenotype of adult NPCs and regulates SOX2 level, a key component of self-renewal. Our results identify PPARγ and PPARβ/δ as regulators of adult neural precursor cell behavior.

## Materials and methods

### Reagents and antibodies

GW0742 and GSK0660 were purchased from Sigma-Aldrich (St. Lois, MO, USA). Rosiglitazone, GW9662 and Bisphenol A diglycidyl ether (BADGE) are from Cayman Chemical Company (Ann Arbor, MI, USA). 5-bromo-2′deoxyuridine is from Sigma-Aldrich (St. Lois, MO, USA). Anti-PPARβ/δ and anti-Myc were obtained from Santa Cruz Biotechnology (Santa Cruz, CA, USA). Anti-PPARγ, anti-GFAP (Glial fibrillary acidic protein), anti-DCX (Doublecortin) and anti-SOX2 are from Cell Signaling Technology (Beverly, MA, USA). Anti-Nestin and anti-EGFR are from Millipore (Billerica, MA, USA), anti-Galactocerebroside C (GalC) was purchased from Sigma-Aldrich (St. Lois, MO, USA), anti-5-bromo-2′deoxyuridine is from Abcam (Cambridge, MA, USA) and anti-β III-Tubulin is from Promega (Madison, WI, USA). Restriction enzymes are all from New England Biolabs (Ipswich, MA, USA). GoTaq Flexi DNA Polymerase and RT-PCR reagents were purchased from Promega (Madison, WI, USA) and Invitrogen (Grand Island, NY, USA). siRNA-PPARβ/δ was purchased from Santa Cruz Biotechonology, siRNA-control and siGlo-Green Transfection Indicator were obtained from Thermo Fisher Scientific, Dharmacon Inc (Lafayette, CO, USA).

### Generation of reporter and expression vectors

Three direct tandems of the peroxisome proliferator response element (PPRE) sequence from the acyl-CoA Oxidase gene were obtained from an original vector, donated by Dr. R. M. Evans (Howard Hughes Medical Institute, The Salk Institute for Biological Studies, La Jolla, CA) (Forman et al., 1995). This sequence was digested with BamHI and HindIII restriction enzymes and was inserted into the commercial vector tkLuc (ATCC, Manassas, VA, USA), specifically, in the 5′ region of the thymidine kinase promoter. This new vector was denominated tkPPRELuc. A luciferase gene was deleted from both vectors (tkLuc and tkPPRELuc) by enzymatic digestion with XhoI and SmaI, and replaced with full-length E. coli βGalactosidase gene, which was obtained from commercial vector pCMVβ (Clontech, Mountain View, CA, USA), by digestion with XhoI and SalI. These vectors were named tkβ Gal and tkPPREβ Gal, respectively.

For Myc-PPARβ/δ vector construction, mouse cDNA of PPARβ/δ was obtained from vector pCMX-PPARβ/δ (donated by Dr. R. M. Evans), by digestion with BamHI and HindIII restriction enzymes, and inserted in pCDNA3-NLS-Myc vector (donated by Dr. Hugo Olguín, Cellular and Molecular Biology Department, P. Catholic University of Chile; Olguin et al., 2007). These restriction sites delete the NLS sequence. For expression and functional analysis of the Myc-PPARβ/δ vector, HEK293 cells were transiently transfected with Lipofectamine-2000 Reagent (Invitrogen, Grand Island, NY, USA). Expression was analyzed by western blot and cellular localization by immunofluorescence. Activity was evaluated for luciferase reporter assay. We did not observe statistical differences in the activity and localization between both vectors (Supplementary Figure 1). All constructs were verified by DNA sequencing.

### Isolation and culture of adult mice NPCs

This study was carried out in strict accordance with the recommendations of the Biosecurity Guide of CONICYT (Comisión Nacional de Investigación Científica y Tecnológica). The Bioethical Committees of Pontifical Catholic University of Chile and University of Chile approved all protocols. We used three-month-old C57bl/6 mice. NPCs culture was prepared according to published protocols (Rietze and Reynolds, 2006; Brewer and Torricelli, 2007). Briefly, the lateral walls of the ventricles, i.e., striatal SVZ, of adult mice brains were dissected, incubated with papain and dissociated with a fire-polished glass pipette in the presence of DNaseI and Ovomucoid (Worthington Biochemical Corporation, Lakewood, NJ). NPCs were cultured as neurospheres in neurobasal medium supplemented with B27 minus vitamin A, 200 mM L-Glutamine (Invitrogen), penicillin/streptomycin (Invitrogen) and 10 ng/ml EGF (Invitrogen). After 7 days in culture, primary neurospheres were dissociated, centrifuged at 110× g, re-suspended and seeded at a density of 10.000 cells/cm^2^ and cultured in the same conditions, in order to generate secondary neurospheres. For all assays we used NPCs seeded as adherent cells. Secondary neurospheres were dissociated into single cells using trypsin-EDTA (Invitrogen), plated onto poly-l-ornithine/laminin (Sigma-Aldrich and Invitrogen, respectively), and cultured as monolayers in a complete medium (neurobasal medium supplemented with B27 minus vitamin A, penicillin/streptomycin, 200 mM L-Glutamine and 10 ng/ml EGF). For differentiation assays, cells were cultivated in neurobasal medium supplemented with B27 minus vitamin A, 200 mM L-Glutamine, penicillin/streptomycin and 0.1% fetal bovine serum, without EGF, in absence or presence of drugs.

### Western blot

Cells were washed with phosphate buffered saline (PBS), lysed and centrifuged at 14.000 xg for 10 min at 4°C. For EGFR detection, lysis buffer was 50 mM Tris-HCl pH 7.5, 150 mM NaCl, 1 mM CaCl2, 1 mM MgCl2 and 0,5% Nonidet P-40 (Garcion et al., 2004). For detection of all other proteins, we used RIPA buffer (20 mM Tris-HCl, 150 mM NaCl, 1 mM EDTA, 1 mM EGTA, 1% Nonidet P-40 and 1% sodium deoxycholate). Both buffers were supplemented with protease and phosphatase inhibitors mixture (Roche Applied Science, Mannheim, Germany). Proteins were resolved by SDS-PAGE and transferred into PVDF membranes. Blots were subsequently incubated with antibodies as follows: Anti-PPARβ/δ (1:1000), anti-PPARγ (1:1000), anti-EGFR (1:1000), anti-Nestin (1:5000), anti SOX2 (1:2000). For detection, horseradish peroxidase-conjugated secondary antibodies (Santa Cruz Biotechnology) were used followed by enhanced chemiluminescence detection (Perkin Elmer Inc., Waltham, MA). Optical density was quantified with ImageJ software.

### Reverse transcriptase polymerase chain reaction (RT-PCR)

Total RNA was isolated from NPCs using Trizol Reagent (Invitrogen. Grand Island, NY, USA) and treated with DNase I (Promega. Madison, WI, USA). 2 μg of RNA was reverse transcribed with SuperScript II Reverse Transcriptase (Invitrogen) and Oligo(dT), according to the manufacturer's instructions. The following primers were used for PCR reactions: forward-PPARβ/δ 5′-GCA GCC TCT TCC TCA ATG AC-3′ and reverse-PPARβ/δ 5′-CCG TCT TCT TTA GCC ACT GC-3′, forward-PPARγ 5′-CTG GCC TCC CTG ATG AAT AA-3′and reverse-PPARγ 5′-ACG TGC TCT GTG ACG ATC TG-3′, Forward-GADPH 5′-TGA CCA CAG TCC ATG CCA TC-3 and reverse-GADPH 5′-GAC GGA CAC ATT GGG GGT AG-3′. The following PCR conditions were used: 94°C for 5 min, followed by 35 cycles of 94°C (30 s), 55°C or 60°C (30 s) and 72°C (30 s). GoTaq Flexi DNA Polymerase and 2 mM MgCl2 were used in all reactions (Promega. Madison, WI, USA). Fragments were analyzed with agarose gel electrophoresis (2%) and SYBR-Safe staining (Invitrogen).

### BrdU incorporation assays

For *in vitro* BrdU-incorporation assays, NPCs were incubated with 10 μM BrdU for 6 h previous to fixation in 4% paraformaldehyde. Samples were incubated 10 min in HCl 2 M, thrice in Sodium Borate Buffer 0,1 M pH 8,5 (10 min each time) and permeabilized/blocked in PBS 0.1% Triton-×100 and 5% normal donkey serum for 30 min. Anti-BrdU antibody (1:500, Abcam. Cambridge, MA, USA) was incubated for 2 h at 37°C. Cy2-conjugated anti-rat IgG was used as a secondary antibody (1:500, Abcam) and incubated for 1 h at room temperature. BrdU-positive cells were evaluated using an Epifluorescent Axioplan Microscope and AxioCam MRm (Zeiss). BrdU positive cells were counted in 15 randomly selected fields from three different coverslips, for each experiment. We used DAPI for total cells count. At least three independent experiments were carried out for each assay.

For *in vivo* BrdU-incorporation assays, mice were intraperitoneally injected with 100 mg BrdU/Kg of animal body weight for 5 days. At day 5, mice were anesthetized and perfused intracardially with PBS, followed by cold 4% paraformaldehyde solution. Brains were collected and post-fixed overnight in 4% paraformaldehyde, followed by 24 h immersion in a 20% sucrose solution. Brains were included in OCT. Coronal sections (30 μm) from SVZ were processed for immunofluorescence. Briefly, slices were incubated 20 min in 0.13 M NaBH4 and washed with PBS, then incubated 10 min in HCl 2 M, 10 min in Sodium Borate Buffer 0,1 M pH 8,5, thrice in TBS and permeabilized/blocked in TBS 0.1% Triton-×100 and 5% normal donkey serum for 30 min. Primary antibodies, anti-BrdU (1:1000) and anti-PPARβ/δ (1:100), were incubated for 48 h at 4°C. Alexa Fluor secondary antibodies (Invitrogen) or Cy2 secondary antibody (Abcam) were incubated for 1 h at room temperature. This protocol was modified from Valero et al. (2005) and Wojtowicz and Kee (2006).

### Immunocytochemistry

Cells were fixed in 4% paraformaldehyde, permeabilized/blocked in PBS-0.1%Triton-X100/5% normal donkey serum for 1 h and incubated in primary antibodies at 4°C overnight. The following primary antibodies were used: anti-PPARβ/δ (1:100), anti-β-Galactosidase (1:1000), anti-Nestin (1:1000), anti-DCX (1/500), anti-SOX2 (1:200) and anti-Myc (1:500). Alexa-Fluor secondary antibodies (1:1000) were incubated 1 h at room temperature. DAPI (Invitrogen) was used for nuclei detection. Samples were examined in an Epifluorescent Axioplan Microscope and AxioCam MRm (Zeiss), or in a Fluoview 1000 Confocal Microscope (Olympus). ImageJ Program was used to analyze and quantify the images.

### Nucleofection of mouse adult NPCs

Nucleofection of adult NPCs was performed, using the mouse NSC NucleofectorTM Kit and optimized protocols provided by the manufacturer (Amaxa Biosystem, Cologne, Germany). Live and dead cells were counted by trypan blue staining in Neubauer hemocytometer after nucleofection and cells were plated onto poly-l-ornithine/laminin coated coverslips in a medium supplemented with growth factors. 24 h after nucleofection, cells were treated with PPAR ligands, for time and concentrations as indicated in the results section. For PPAR reporter assay, images were acquired with an Epifluorescent Axioplan Microscope and AxioCam MRm (Zeiss). Cells were delimited and βGalactosidase fluorescence was quantified using ImageJ.

### Transfection of siRNA

Cells were seeded onto poly-l-ornithine/laminin coated coverslip in a complete medium supplemented with EGF. Cells were co-transfected with siGlo-Green/siRNA-Control or siGlo-Green/siRNA-PPARβ/δ using DharmaFECT 3 transfection reagent (Dharmacon), according to the manufacturer's instructions. Transfected cells were maintained in complete medium with EGF for 48 h, the medium was replaced every day. Silencing of PPARβ/δ was evaluated by western blot and followed by anti-SOX2 immunofluorescence. Images were taken with an Epifluorescent Axioplan Microscope and AxioCam MRm (Zeiss). SOX2 fluorescence was quantified in siGloGreen positive cells. As SOX2 is a nuclear factor, nucleus was delimited in DAPI positive area and fluorescence of SOX2 was quantified in this region using ImageJ program.

### Statistical analysis

Mann Whitney Test and One-Way ANOVA-Bonferroni were used to analyze the statistical differences of means. *p* < 0.05 (95% confidence intervals) was considered significant. Prism Program was used for all analysis. Values are expressed as mean ± standard error of the mean (SEM).

## Results

### PPARβ/δ and PPARγ are present in mouse adult SVZ NPCs and activities of these receptors are inducible by exogenous ligands

**Figure 1:**
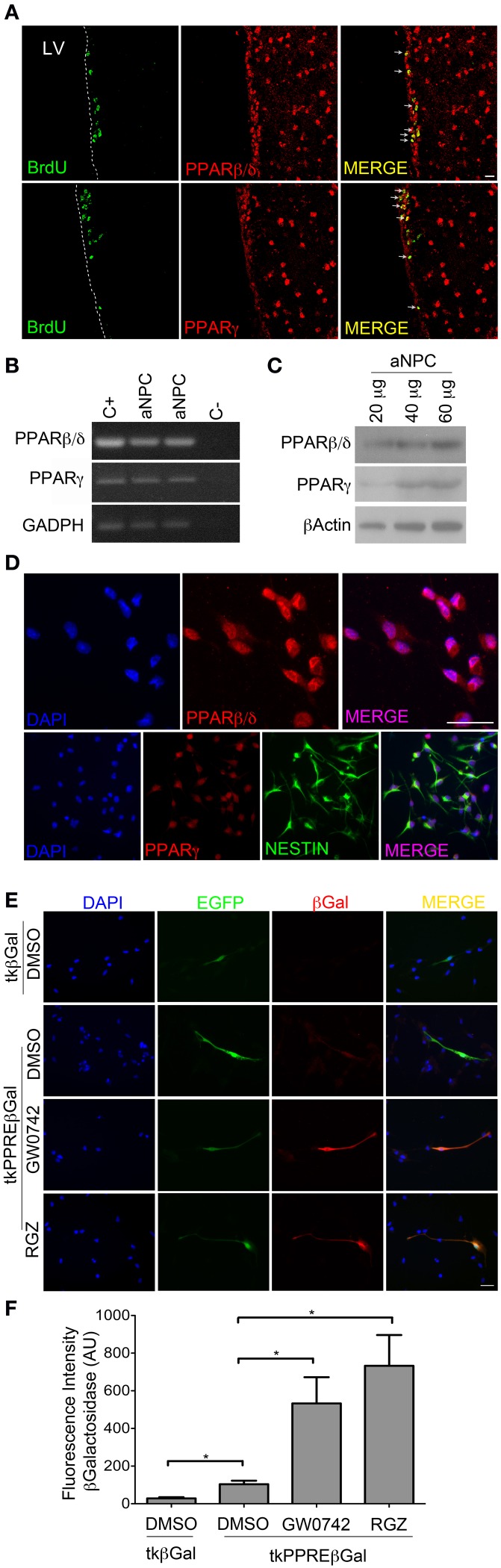
**PPARβ/δ and PPARγ are expressed in adult NPCs and their transcriptional activity is inducible by exogenous ligands. (A)** Adult mice were injected with 100 mg BrdU/Kg animal body weight for labeling of proliferative cells. Brain coronal sections of 30 μm were immunostained with anti-PPARβ/δ or anti- PPARγ (red) and anti-BrdU (green) antibodies. Samples were analyzed by confocal microscopy. **(B)** RT-PCR for PPARβ/δ and PPARγ: RNA was extracted from SVZ NPCs. Neocortical and adipose tissues from adult mice were used as positive controls (C+), respectively. **(C)** Western blots anti-PPARβ/δ and PPARγ. **(D)** Immunofluorescence anti-PPARβ/δ and PPARγ. **(E,F)** *In situ* transcriptional activity assay: Adult NPCs were transiently co-transfected with pEGFP-C1 reporter vector and tkPPREβ Gal (PPAR activity transcriptional reporter vector) or tkβ Gal (empty vector). Cells were treated with GW0742 1 μM, Rosiglitazone 1 μM (RGZ) or DMSO (vehicle) for 24 h. Immunofluorescence anti-βGalactosidase (β Gal) was performed. Fluorescence intensity was quantified by ImageJ program. Graphic in **(E)** represent the mean fluorescence intensity ± s.e.m (* *p* < 0.05). AU, Arbitrary Units.

In order to evaluate if PPARβ/δ and PPARγ are expressed in proliferating NPCs in the SVZ, adult mice were injected for 5 days intraperitoneally with BrdU followed by immunostaining on coronal brain sections. We observed that both PPARβ/δ and PPARγ are expressed in BrdU positive cells in the SVZ of adult mice, as detected by co-labeling with BrdU immunostaining (Figure 1A). Both PPARs have a nuclear expression. As previously described (Braissant et al., 1996), PPARβ/δ expression is also observed in striatum and cortex. In order to establish a possible role of PPARs in these progenitors, we prepared primary cultures of NPCs from the SVZ of adult mice. PPARβ/δ and PPARγ are expressed in NPCs *in vitro* (Figures 1B,C) displaying mainly a nuclear localization, as revealed by immunofluorescence (Figure 1D).

Next, we sought to find out if PPARs are transcriptionally active *in vitro* and if their activity is inducible by exogenous ligands in these cells. In order to detect PPAR transcriptional activity, we generated βGalactosidase reporter vectors, called tkPPREβ Gal and tkβ Gal (see Material and Methods Section), which allowed us to detect PPAR activity *in situ* by immunofluorescence. This method was applied due to low plasmid transfection efficiency of NPCs (15.53 ± 5.43% for Nucleofection and 4.40 ± 1.71 for Lipofectamine-2000 Reagent; Mean ± SD). To be able to identify cells carrying βGalactosidase vectors and subsequently quantify fluorescence intensity of βGalactosidase on these cells, we performed co-transfection with an EGFP vector. Co-transfection efficiency in NPCs in our system was 56.85 ± 14.49% (mean ± SD), as evaluated by co-nucleofection of EGFP and dsRed-N1 vectors. Transfected cells were treated for 24 h with a PPARβ/δ agonist (GW0742) or vehicle (DMSO). In control condition, we observed that NPCs transfected with tkβ Gal presented a 3.8-fold lower intensity of fluorescence vs. cells transfected with tkPPREβ Gal. When NPCs transfected with the tkPPREβ Gal vector were treated with GW0742, we detected a 5-fold increase in the fluorescence intensity with respect to cells treated with DMSO (Figures 1E,F). In line with this observation, Rosiglitazone (PPARγ agonist) increased the fluorescence intensity 7 times with respect to the control.

These results reveal endogenous transcriptional activity of PPARs in adult SVZ-NPCs, and also that their activities can be induced by exogenous ligands.

### PPARβ/δ and PPARγ agonists induce proliferation of NPCs

**Figure 2:**
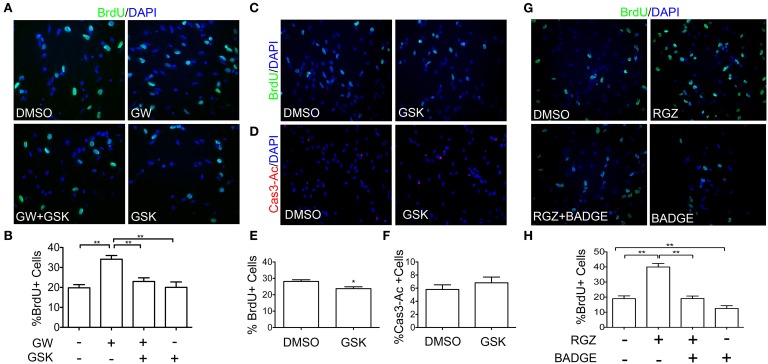
**PPARβ/δ and PPARγ ligands regulate cell proliferation. (A)** NPCs cultures were treated with a vehicle (DMSO), 1 μM GW0742 (GW; PPARβ/δ agonist), 5 μM GSK0660 (GSK; PPARβ/δ antagonist) or both, for 24 h, in the presence of 10 ng/ml EGF. BrdU was added to the cultures followed by anti-BrdU immunodetection. Graphic in **(B)** represents the mean percentage of BrdU positive cells ± s.e.m. of three independent experiments *N* = 3; ^**^p < 0.01. **(C)** Cells were treated with 5 μM GSK0660 or a vehicle (DMSO) for 48 h in the presence of 10 ng/ml EGF. BrdU was added to the cultures 6 h prior to fixation. Immunostaining with anti-BrdU was performed. The Graph in **(E)** represents the mean percentage of BrdU positive cells ± s.e.m. (^*^p = 0.012, Mann Whitney Test). **(D)** Cells were treated with 5 μM GSK0660 or a vehicle (DMSO) for 48 h in the presence of 10 ng/ml EGF. Immunofluorescence anti-Activated Caspase3 (Cas3-Ac) was performed to identify apoptotic cells. The Graph in **(F)** represents the mean percentage of Caspase3-Ac positive cells ± s.e.m. of three independent experiments. No statistical differences were observed (*N* = 3; *p* = 0.4338). **(G)** Adult NPCs were treated with a vehicle (DMSO), 1 μM Rosiglitazone (RGZ; PPARγ agonist), 50 μM of BADGE (PPARγ antagonist) or both, for 24 h, in the presence of 10 ng/ml EGF. Immunodetection with anti-BrdU is shown. The Graph in **(H)** represents the mean percentage of BrdU positive cells ± s.e.m. of three independent experiments *N* = 3; ^**^p < 0.01.

Having established that PPARβ/δ is expressed in NPCs and that this activity is inducible by exogenous ligand *in vitro*, we next evaluated if PPARβ/δ plays a role in NPCs proliferation by performing a BrdU incorporation assay. NPCs were seeded as a monolayer in presence of 10 ng/ml of EGF and treated with PPARβ/δ ligands. A BrdU pulse was performed 6 h previous to the experiment end-point. Cell proliferation showed an increase of almost 30% in cultures treated with PPARβ/δ agonist (GW0742) for 24 h and this effect was reverted by incubation with a PPARβ/δ antagonist (GSK0660), suggesting that this increase is indeed PPARβ/δ-dependent (Figures 2A,B). We should note that we did not observe any effect of antagonist treatment in basal conditions in a 24 h treatment. Nevertheless, when cells were treated with the same antagonist for a longer time period (48 h), we did observe a small but statistically significant decrease in the percentage of BrdU positive cells (32.12 ± 0.66 v/s 27.28 ± 1.04; *p* = 0.003) (Figures 2C,E). We did not see differences in the percentage of activated caspase-3, as evaluated by immunofluorescence (Figures 2D,F), indicating that apoptosis does not account for this decrease. Rosiglitazone, a PPARγ agonist, also induced an increase of proliferation in NPCs treated for 24 h, an effect that is reverted by a PPARγ antagonist (BADGE). Unlike the PPARβ/δ antagonist, BADGE is able to decrease basal level of BrdU incorporation at 24 h (Figures 2G,H).

### PPARβ/δ antagonist drives differentiation of NPCs even in presence of EGF

**Figure 3:**
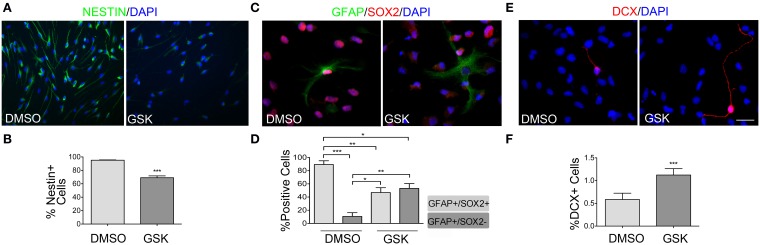
**PPARβ/δ antagonist diminished precursor phenotype**. Adult NPCs were treated with 5 μM GSK0660 or the vehicle (DMSO) for 48 h in the presence of 10 ng/ml EGF. Different markers were evaluated by immunofluorescence. Quantification of percentage in different conditions was performed and shown in the graphs. **(A)** Immunofluorescence anti-Nestin. **(C)** Immunofluorescence anti-GFAP/SOX2. In GSK0660 treated cells we detected a change in the percentage of GFAP+/SOX+ and GFAP+/SOX2− cells, as observed in the graph. Control shows a representative image of GFAP+/SOX2+ cells, the predominant population. GSK0660 shows a representative image of GFAP+/SOX2− cells, whose population increased in this condition. In both conditions, light gray bars represent GFAP+/SOX2+ cells and dark gray bars represent GFAP+/SOX2− cells. Percentages expressed are in respect to the total GFAP+ cells. **(E)** Immunofluorescence anti-DCX. Graph in **(B,D,F)**, represent the mean percentage of positive cells ± s.e.m. of three independent experiments. Bars correspond to 20 μm. Mann Whitney Test was applied in **(B,F)** and One-way ANOVA-Bonferroni Test was applied in **(D)**. (^*^p < 0.05, ^**^p = 0.01 to 0.001, ^***^p < 0.001).

In addition to regulating proliferation, PPARβ/δ could also act by maintaining the NPCs pool. In order to evaluate this hypothesis, NPCs, seeded as single cells over poly-L-ornithine/laminin, were treated with the PPARβ/δ antagonist for 48 h, in presence of 10 ng/ml EGF. A precursor phenotype was evaluated for Nestin, GFAP and SOX2 expression. In control samples, over 95% of the cells were positive for SOX2 and Nestin expression. Nestin positive cells showed a relative homogenous morphology, with two or three thin processes of different lengths (Figure 3A). In contrast, a significant decrease in Nestin positive cells in cultures treated with the drug was observed (95.10 ± 0.55% vs. 69.06 ± 2.74%; *p* < 0.0001) (Figures 3A,B). In addition, a decrease in fluorescence intensity was also observed as shown in Figure 3A. GFAP during late cortical embryogenesis and in adult SVZ is considered a marker of many terminally differentiated astrocytes, but has also been reported as a NSC/Radial glia marker. Therefore, in order to check if GFAP labeling is related to NPCs or to mature astrocyte, we co-labeled the cells with the stem cell marker SOX2. In control conditions, 89.41 ± 5.66% of the GFAP positive cells are SOX2+/GFAP+ vs. 10.60 ± 5.66% SOX−/GFAP+; cultures treated with the antagonist (5 μM GSK0660) changed these percentages to 46.90 ± 7.34% vs. 53.10 ± 7.34%, for SOX2+/GFAP+ and SOX2−/GFAP+, respectively (Figures 3C,D). In agreement with these results, we also observed a clear change in the morphology of these cells. In the presence of the PPARβ/δ antagonist, GFAP positive cells presented a greater number of processes and the projections were thicker, a suggestive morphology of mature astrocytes (Figure 3C). In addition, treatment with this inhibitor resulted in an increase in the percentage of neurons in the culture. It should be noted that a spontaneous but minimal neuronal differentiation is observed in presence of growth factors when monolayer cultures are maintained for 3 days or more in normal conditions (medium supplemented with 10 ng/ml EGF), as evidenced by a 0.50 ± 0.14% of DCX positive cells. But in cultures treated with the PPARβ/δ antagonist, the percentage increased to 1.12 ± 0.14% (*p* < 0.0001) and some cells even displayed morphologies characteristic of mature neurons in contrast with DCX positive cell morphologies observed in the controls (Figures 3E,F). Finally, regarding oligodendrocytes, neither in the controls nor after treatments, we were able to observe changes, as evaluated by an immunofluorescence anti-GalC (data not shown). From these results, it clearly emerges that PPARβ/δ participates in the maintenance of the precursor phenotype.

### PPARβ/δ regulates SOX2 level in NPCs *in vitro*

**Figure 4:**
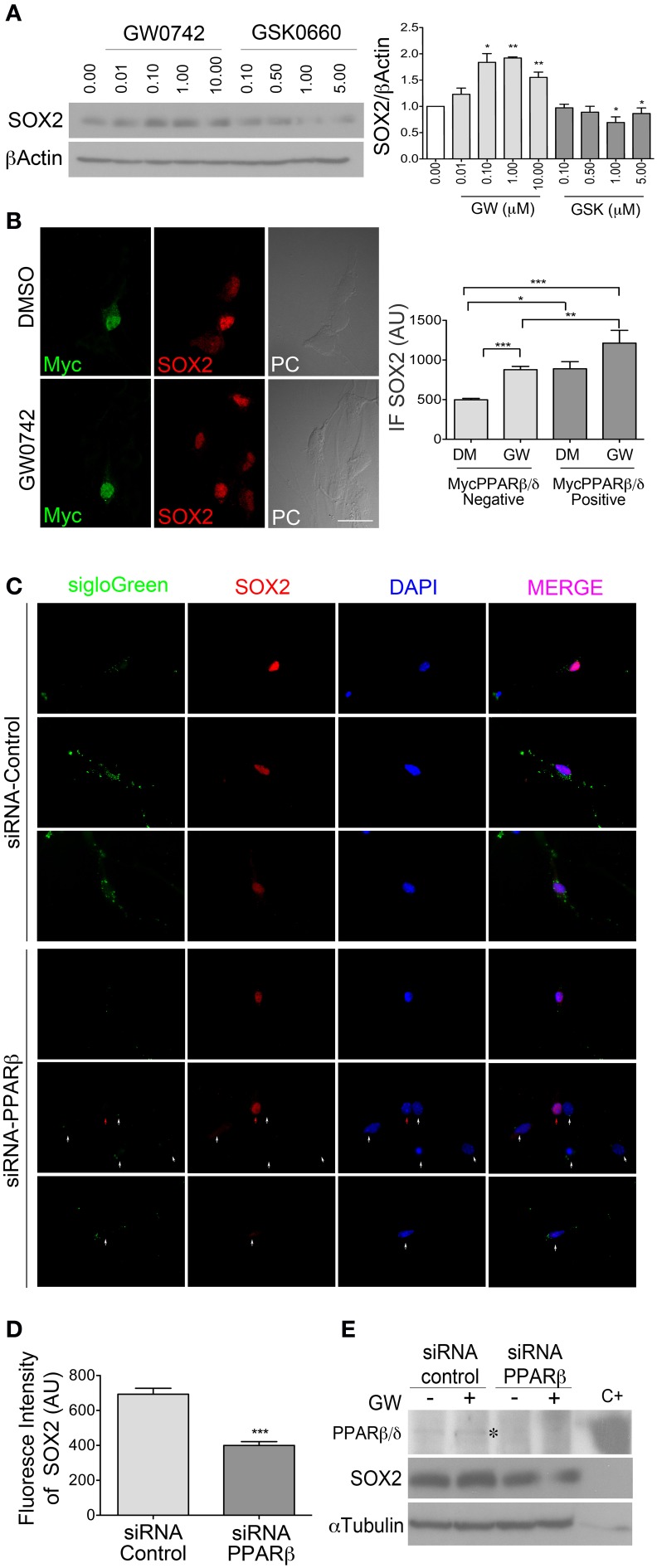
**PPARβ/δ regulate SOX2 level in adult NPCs**. **(A)** NPCs were treated for 24 h at different concentrations of GW0742 or GSK0660 as indicated, in the presence of 10 ng/ml EGF. Total proteins were analyzed by western blot for anti-SOX2. Graph represents the mean ± s.e.m. of three independent experiments. One-Way ANOVA-Bonferroni Test was applied. **(B)** Myc-PPARβ/δ expression vector was transiently transfected. Cells were treated with 1 μM GW0742 (agonist) or a vehicle (DMSO) for 24 h.Immunofluorescence anti-SOX2/anti-Myc was realized. SOX2 fluorescence intensity was quantified in both Myc positive and negative cells. Graph represents the mean ± s.e.m. of three independent experiments.One-Way ANOVA-Bonferroni Test was applied. **(C)** Adult NPCs were transiently co-transfected with siGLO-Green and siRNA-PPARβ/δ or siRNA-control, in the presence of 10 ng/ml of EGF. SOX2 fluorescence intensity was quantified in siGLO-Green positive cells. Representative images of cells observed and quantified are shown. White arrows shows siGLO-Green positive cell. Red arrow shows a siGLO-Green negative cell. Graph in **(D)** represents the mean ± s.e.m. **(E)** NPCs were transfected with siRNA-PPARβ/δ or siRNA-control, under the same condition as in **(C)**. Cells transfected were treated with 1 μM GW0742 (GW) for 24 h in the presence of 10 ng/ml of EGF. Western blots anti-PPARβ/δ and anti-SOX2, Total protein extract from HEK293 cells transiently transfected with pCMX- PPARβ/δ was used as a positive control (C+). (^*^p < 0.05, ^**^p = 0.01–0.001, ^***^p < 0.001, Mann Whitney Test). DM, DMSO; GW, GW0742; AU, Arbitrary Units.

SOX2 is an important regulator of NPCs self-renewal and since we observed a decrease in its expression level after treatment with the PPARβ/δ antagonist by immunostaining (Figures 3C,D), we were interested in evaluating if this receptor regulates SOX2 directly. Indeed, PPARβ/δ antagonist decreased SOX2 expression below the basal level, acting in a dose dependent manner, whereas the agonist increased SOX2 level (Figure 4A). In order to characterize the PPARβ/δ overexpression effect, we took two issues into consideration: 1) adult NPCs present low transfection efficiency, complicating the analysis by western blot and 2) PPARβ/δ is already expressed in basal conditions in adult NPCs. To overcome these difficulties, we generated a PPARβ/δ expression vector with an additional tag (Myc-PPARβ/δ), allowing us thereby to identify individual transfected cells that were overexpressing PPARβ/δ. The expression and functionality of the Myc-PPARβ/δ vector were evaluated first in a Hek293 cell line. We did not observe any change in cell behavior and activity of Myc-PPARβ/δ in comparison to PPARβ/δ without the tag (Supplementary Figure 1).

NPCs were transfected with Myc-PPARβ/δ and ectopic expression of PPARβ/δ in the cells was evaluated by anti-Myc immunofluorescence. Transfected cells were treated with PPARβ/δ agonist (GW0742) or vehicle (DMSO) for 24 h, followed by an anti-SOX2/anti-Myc co-immunofluorescence. The fluorescence intensity of SOX2 was evaluated in both Myc-PPARβ/δ positive and negative cells, in presence or absence of the agonist. In control conditions (DMSO) we were able to observe a 1.78-fold increase in the SOX2 fluorescence intensity in Myc-PPARβ/δ positive cells with respect to Myc-PPARβ/δ negative cells. When cells were cultured in the presence of PPARβ/δ agonist (GW0742), the fluorescence intensity increased in both conditions, Myc-PPARβ/δ negative and positive cells, but the increment in Myc-PPARβ/δ positive cells were much greater (1.76-fold vs. 2.44-fold, in Myc-PPARβ/δ negative and positive cells, respectively, both treated with GW0742 compared to Myc-PPARβ/δ negative cells treated with the vehicle). We did not observe statistical differences between Myc-PPARβ/δ positive cells treated with GW0742 or vehicle (Figure 4B).

### PPARβ/δ is necessary for SOX2 expression in mouse adult NPCs from the SVZ

In order to establish if PPARβ/δ is necessary for SOX2 maintenance, we subsequently did a knock down of this factor in NPCs by using siRNA, co-transfecting either siGlo-Green with siRNA-PPARβ/δ or the siRNA-control. Transfection efficiency was higher than 80% (Percentage of siGLO-Green positive cells). Interestingly, we observed an average decrease of 0.6-fold in the fluorescence intensity of SOX2 in cells transfected with siRNA-PPARβ/δ with respect to the control (*p* < 0.0001; Figures 4C,D). Next, NPCs were transfected with siRNA-PPARβ/δ or siRNA-control and treated with 1 μM GW0742 for 24 h in the presence of 10 ng/ml of EGF, followed by anti-PPARβ/δ and anti-SOX2 Western blots. An expected increase of SOX2 level was observed in control cells treated with the agonist, as already observed (Figures 4A,E). Moreover, PPARβ/δ knockdown decreased SOX2 level while PPARβ/δ agonist in this condition was not able to increase the level of SOX2 (Figure 4E).

Thus, our results show that PPARβ/δ contributes toward the maintenance of the precursor phenotype and regulates SOX2 level in adult NPCs. Since PPARβ/δ is a transcription factor that regulates the expression of its target genes by recognizing specific sequence denominated PPRE (PPAR response element) in the regulatory region, we analyzed the mouse *Sox2* gene (Gene ID 20674) for the presence of possible response elements in the promoter region. Note that the Mat Inspector Program revealed two putative PPREs in the positions −203 to −225 (GTCTTGGTGCTGTTTACCCACTT) and −244 to −266 (CCGTTTTCAGCAACAGGTCACGG), in respect of the transcription site, suggesting a direct transcriptional regulation of *Sox2* by PPARβ/δ. Further *in vitro* studies will be required to confirm this *in silico* result.

### PPARγ and PPARβ/δ regulate EGFR level in NPCs

As already shown, PPARβ/δ regulates SOX2 level in NPCs *in vitro*. SOX2 is an important transcription factor necessary in the maintenance of undifferentiated phenotypes of these cells. Interestingly, it generates a positive feedback loop with EGFR, also involved in self-renewal of NPCs (Hu et al., 2010). Additionally, Wada et al. reported recently that PPARγ regulates EGFR level in embryonic mouse NPCs (E13.5–E14.5), increasing thereby their cellular viability. We therefore decided to evaluate if PPARγ has the same effect on adult NPCs as on embryonic ones and, in addition, if PPARβ/δ is also able to regulate EGFR level.

**Figure 5:**
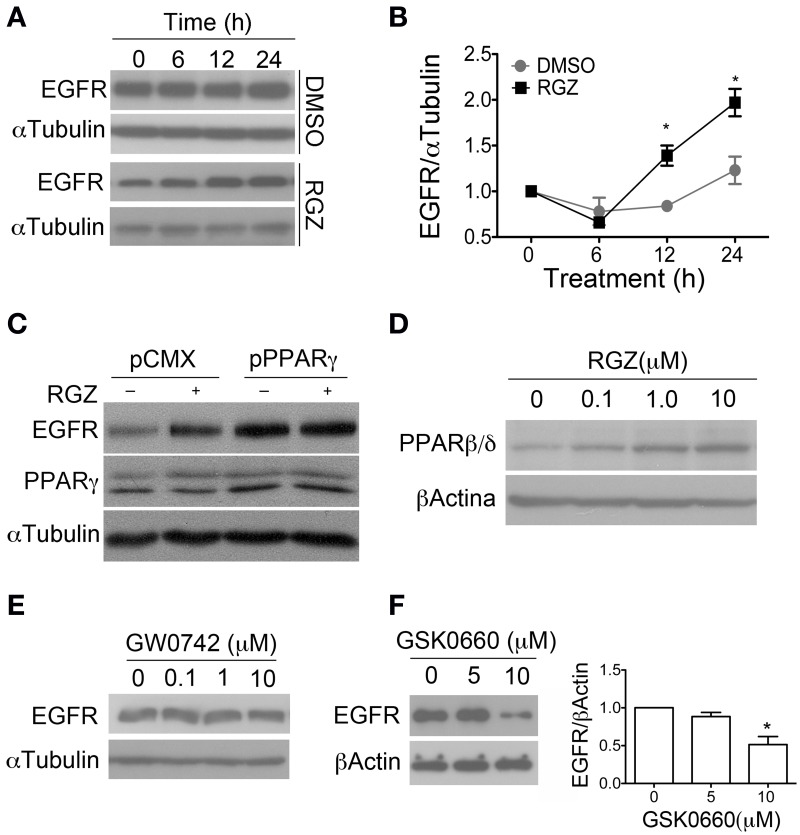
**PPARγ regulate EGFR level in adult NPCs**. **(A)** NPCs were treated with 1 μM rosiglitazone (RGZ) or a vehicle (DMSO), for the times indicated. Proteins were analyzed by western blot anti-EGFR. αTubulin was used as a loading control. In **(B)** graph represents the mean ± s.e.m. of three independent experiments (One-way ANOVA-Bonferroni test; ^*^ *p* < 0.05). **(C)** NPCs were transfected with PPARγ1 expression vector (pPPARγ) or empty vector (pCMX) and treated with 1 μM Rosiglitazone (RGZ) or a vehicle (DMSO) for 24 h. Total proteins were analyzed by western blot for anti-EGFR. Western blot with anti-PPARγ is also shown. Only PPARγ overexpression increases EGFR level up to 3-fold. **(D)** Adult NPCs were treated for 24 h with RGZ at the concentrations indicated in the figure. PPARβ/δ level were analyzed by western blot. Adult NPCs were treated with GW0742 **(E)** and GSK0660 **(F)** at different concentrations. Level of EGFR was evaluated by western blot.

We observed that the PPARγ agonist (Rosiglitazone) increased EGFR level at 12 h and this effect lasted up to 24 h (Figures 5A,B). This effect is reverted by the antagonist (BADGE), suggesting PPARγ dependence. Moreover, an increase in the EGFR level at short times (15–30 min) in respect to time zero was also observed in both control and treated cells, consistent with data reported by Hu et al. and probably due to the addition of EGF to the culture (Hu et al., 2010). Nevertheless, we did not observe statistically significant differences between treatments at these short times (data not shown). Finally, when PPARγ was overexpressed in adult NPCs, concomitant to the higher proliferation rate of NPCs cultures, we found increased EGFR level, even in basal conditions (Figure 5C). Remarkably, 24 h treatment with the agonist also increased PPARβ/δ protein level in a concentration-dependent manner indicating a possible collaborative function between PPARs (Figure 5D).

With regard to the PPARβ/δ function over EGFR level, NPCs were treated for 24 h with PPARβ/δ agonist (GW0742) and EGFR protein was evaluated by western blot. We did not observe any difference between treatment and control (Figure 5E). However, when cells were treated with the antagonist (GSK0660) for 48 h at a concentration of 10 μM, protein level of EGFR was clearly diminished (Figure 5F).

We conclude that PPARγ regulates EGFR level in adult NPCs. Additionally, that the inhibition of PPARβ/δ alone is able to modulate EGFR level, with a different kinetic action regarding PPARγ, suggesting an indirect effect of PPARβ/δ over EGFR. Finally, PPARγ regulates PPARβ/δ level suggesting a cooperative effect in the maintenance of NPCs undifferentiated phenotype.

## Discussion

In this study we reveal a new role of PPARs acting in maintenance of adult NPCs undifferentiated phenotype *in vitro*. We demonstrate the presence of PPARβ/δ and PPARγ in proliferative cells in the SVZ *in situ* and describe their mainly nuclear expression pattern in NPCs cultures. PPARs appear to have endogenous activity in these cells and their activity can be induced by exogenous ligands, as shown by pharmacological activation, thereby allowing a detailed functional analysis of PPARs in NPCs. Our results, therefore, imply an endogenous activity of PPARs regulating NPCs behavior.

PPARβ/δ is a transcription factor expressed, with high level in selected tissues such as skin, fat and brain. Over 90% of PPARβ/δ-null mice are not viable and die at early embryonic stages, mainly due to placental disorders (Barak et al., 2002). The surviving mutant mice have disorders in myelination of the central nervous system and decreased adipose mass. In adipose tissue and skin, PPARβ/δ has a role in apoptosis, proliferation and differentiation (Peters et al., 2000; Di-Poi et al., 2005). Here we show that PPARβ/δ contributes to maintain undifferentiated phenotype and regulates proliferation of adult NPCs *in vitro*. These two important functions are involved in self-renewal of NPCs, an essential property *in vivo* to maintain the NPCs pool in specific regions of the adult brain.

One of the most important factors involved in NPCs self-renewal is SOX2. Adult Sox2-KO mice present reduced proliferation and neurogenesis in SVZ and Hippocampus (Ferri et al., 2004). Interestingly, we show that PPARβ/δ is a regulator of SOX2 levels. Moreover, PPARβ/δ is necessary for SOX2 expression. Consistent with this notion, we found two putative PPRE elements in the promoter region of this factor, suggesting a direct role in its transcriptional control. SOX2 is not only necessary for maintenance of the embryonic and adult neural stem/precursor phenotype, it also has been demonstrated that this factor is essential for inner cell mass embryonic stem cells, as its ablation causes early embryonic lethality (Avilion et al., 2003). Moreover, SOX2 is one of four-transcription factors necessary for induction of fibroblast to pluripotent cells (Takahashi et al., 2006; Takahashi and Yamanaka, 2006). Thus, understanding the mechanism and identifying factors involved in the regulation of SOX2 expression is a relevant topic.

We described that inhibition of PPARβ/δ decreases the level of SOX2, but also changes the phenotype of these cells, inducing differentiation. Adult NPCs culture display high percentage of SOX2 and Nestin positive cells (~95%). The evaluation of the PPARβ/δ activation effect on population phenotypes is technically difficult, but it would be interesting to address if ectopic expression of SOX2 and even PPARβ/δ activation on specific-phenotype committed precursor (to neuron, astrocyte or oligodendrocyte; SOX2−/Nestin−) can return them into an earlier precursor stage (SOX+/Nestin+).

Interestingly, SOX2 presents a positive feedback with EGFR in embryonic NPCs involved in self-renewal (Hu et al., 2010). EGFR is a membrane receptor of several extracellular ligands, including EGF, but also can be transactivated by external inputs via interaction with other pathways, such as SHH, acting as a nodal mediator in the control of cellular behavior (Reinchisi et al., 2013). *In vivo*, EGFR is expressed in the SVZ, mainly in activated neural stem cells (B-cells) and in the transit-amplifying C-cell population (Doetsch et al., 2002; Pastrana et al., 2009). This population, in response to EGF, increases its proliferation and maintains the multipotent characteristic (Doetsch et al., 2002). *In vitro*, maintenance of adult mice NPCs cultures requires EGF; deprivation of this ligand in the medium induces spontaneously differentiation to neurons, astrocyte and oligodendrocytes. Interestingly, PPARβ/δ is a mediator of the EGF-EGFR pathway in skin, one of the organs where this transcription factors is highly expressed. In HaCat keratinocytes, EGF induces up-regulation of PPARβ/δ expression, increased DNA binding and promotes its transcriptional activity. Conversely, PPARβ/δ knockdown leads to decreased EGF-mediated cell proliferation (Liang et al., 2008b). Cells pretreated with a PPARβ/δ agonist, show a smaller percentage of apoptosis induced by TNF-α and an increased protective effect of EGF (Liang et al., 2008a), suggesting a PPARβ/δ-dependent mechanism in EGF-stimulated cell proliferation and survival. Furthermore, PPARβ/δ is involved in cell proliferation of other cells types such as endothelial cells (Piqueras et al., 2007), pre-adipocytes (Hansen et al., 2001), human breast, prostate, gastric and hepatocellular carcinoma cells (Glinghammar et al., 2003; Stephen et al., 2004; Nagy et al., 2011). Our results show that a PPARβ/δ antagonist is able to decrease level of EGFR and proliferation of cells after 48 h of treatment, but no increase was observed when cells were treated with the agonist. Although at this moment we have no explanation for this last result, we can not rule out that the effect in this context requires a longer period of analysis. Additional approaches addressing the relationship of PPARβ/δ and EGFR signaling pathways will have to be undertaken in order to obtain a better understanding of how and when these pathways interact to control NPCs behavior.

Our data suggest that regulation of EGFR level and proliferation of cells by PPARβ/δ antagonists can be consequences associated with changes in the phenotype of the NPCs, as we show that antagonists induce differentiation, even in presence of EGF. In addition, the absence of changes in EGFR level after activation of PPARβ/δ seem to be contradictory with the evidence of the positive feedback between EGFR and SOX2 in neural precursor cells, but this could just be due to the timing evaluated in this study. Also, it might be possible that PPARβ/δ acts as a mediator of the EGF-EGFR pathway modulating self-renewal function through the regulation of SOX2, however this hypothesis requires more experimental evidence to be confirmed.

On the other hand, we also show that PPARγ regulates EGFR level as well as proliferation of adult NPCs. Rosiglitazone, a PPARγ agonist, increases the level of EGFR at 12 h post-treatment, which is maintained at least until 24 h. Overexpression of this transcription factor is sufficient to induce an increase of EGFR level. Concomitant with this effect, ligands of PPARγ regulate proliferation of adult NPCs, indicating that this PPAR isoform is involved in the control of proliferation and maintenance of NPCs, probably acting through the transcriptional control of EGFR. We observed that cultures deprived of EGF in order to induce differentiation of NPCs and treated with Rosiglitazone, present a higher number of Nestin positive cells at 48 h of treatment. Even if the effect of Rosiglitazone is not sufficient to maintain the cells in undifferentiated phenotype after 72 h of EGF deprivation (differentiation assays; data not shown), we observe a slower rate of differentiation. Wada et al. reported an increase of cell viability in embryonic NPCs treated with rosiglitazone (PPARγ agonist) and interestingly, stated that NPCs obtained from embryonic PPARγ-KO mice (E13.5) have decreased size and numbers. Moreover, this group reported regulation of EGFR by PPARγ, and suggested that regulation of proliferation by PPARγ could be mediated by regulation of EGFR (Wada et al., 2006). Consistent with these data, Burrows et al. showed that level and density of EGFR are important for cell fate and proliferation of NPCs, and that cell response depends, at least partially, on ligand concentration (Burrows et al., 1997). However, our observations are in disagreement with results from *in vitro* experiments published by Morales-Garcia et al. (Morales-Garcia et al., 2011). They proposed that pioglitazone and rosiglitazone, PPARγ agonists, induce differentiation of NPCs *in vitro*. One explanation for these opposite findings could be the different model systems used (rat vs. mouse), but also, and more importantly, the concentration of the drugs used in the study. Morales-Garcia used 30 μM of rosiglitazone in a pre-treatment of 7 days, previous to the differentiation assay (Morales-Garcia et al., 2011). Rosiglitazone binds to the PPARγ ligand-binding domain with a Kd of 43 nM and activated a luciferase reporter vector with an approximately EC50 of 0.1 uM (Lehmann et al., 1995) and pioglitazone and rosiglitazone at a concentration 10 μM, induce activation of PPARα, in COS-1 cells (Sakamoto et al., 2000). Moreover, Wada et al. showed that in embryonic neurospheres, rosiglitazone has a biphasic effect. At low concentrations, this agonist induces proliferation and maintenance of a stem/precursor state, but at a concentration 30 μM or more, rosiglitazone induces apoptosis (Wada et al., 2006). However, Morales et al., showed an interesting effect *in vivo*, with an increase of proliferation in the SVZ that consequently leads to increase in neurogenesis, revealing that the size of the NSC population in the SVZ is important in the regulation of neurogenesis. Thus, the *in vivo* pharmacological activation of PPARγ, by thiazolidinedione, could be an early event in the increase of the NPCs population with the consequent increase in neurogenesis (and as such not necessary a direct induction of differentiation). Accordingly, when Ghoochani et al. evaluated the expression of PPARγ in the process of differentiation from embryonic stem cells to neurons, they observed that the level of PPARγ were increased in the formation of NPCs but then decreased in terminal neuronal differentiation (Ghoochani et al., 2012). Added to that, pharmacological treatment with ligands at different times of the differentiation process revealed that inactivation of PPARγ at early stage in mESC decreased the formation of NPCs, and later of neurons and astrocytes (Ghoochani et al., 2012).

Our results demonstrate that PPARs seem to be active endogenously, at least *in vitro*. Thus, an interesting question is to answer which endogenous ligands can be mediating the effect of PPARs in neural precursor cells. PPARs are described to be nuclear transcription factors and sensors of the lipid metabolism. Known endogenous PPAR ligands in the brain include omega-3 fatty acids, docosahexaenoic acid (DHA) and eicosapentanoic acid (EPA). These ligands have been described to have neuroprotective effects (reviewed in Michael-Titus and Priestley, 2014) and to be involved in proliferation and differentiation of NPCs (Dyall et al., 2010; Sakayori et al., 2011). Another interesting family of endogenous ligands of PPARs is the endocannabinoids (Reviewed in Pistis and Melis, 2010). Activation of the endocannabinoid system in NPCs by a synthetic ligand increases cellular proliferation as well as both number and size of neurospheres (Aguado et al., 2005). CB1 receptor, a key component of this system, was described to be required for neurospheres formation (Aguado et al., 2005) and even more, for neurogenesis *in vivo* (Jin et al., 2004). The roles described for all these ligands in neural precursor cells, positions them as the main candidates to mediate PPAR functions in NPCs, but further investigation will be required to identify specific ligands associated to PPAR function in NPCs.

Finally, we observed that PPARγ agonist is also able to increase PPARβ/δ level, suggesting a possible cooperative effect of these two isoforms of PPAR, which suggest a possible mechanism in NPCs where PPARγ and PPARβ/δ could be mediators between EGFR and SOX2 positive feedbacks, contributing thereby to maintain an undifferentiated phenotype in adult SVZ-NPCs.

## Author contribution

CB, MB designed the research. CB, MB and VP analyzed data. CB and CA performed the research. CB and VP wrote the paper.

### Conflict of interest statement

The authors declare that the research was conducted in the absence of any commercial or financial relationships that could be construed as a potential conflict of interest.

## Supplementary material

Supplementary Figure 1 **pMyc-PPARβ. (A)** Double anti-Myc/anti-PPARβ immunofluorescence. Representative confocal microscopy images are shown. **(B)** Myc-PPARβ expression was evaluated by western blot with anti-PPARβ and anti-Myc antibodies. pPPARβ was used as a control of wild type protein overexpression. pCDNA3-Myc and pCMX are empty vectors for pMyc-PPARβ and pPPARβ, respectively. **(C–E)** Functional analysis of pMyc-PPARβ by luciferase reporter assay. Cells were transfected with the vectors as indicated plus PPRE luciferase reporter vector and pCMXβ as a control. Luciferase activity was measured and shown in respect to control vector (pCMVβ). **(C)** Cells were treated with PPARβ/d agonist (GW, 1 μM) or vehicle (DMSO) for 24 h. **(D)** Different concentrations of GW0742 were used to evaluate activity and to compare both vectors. **(E)** Cells were pre-treated with antagonist (GSK) for 1 h and then co-treated with PPARβ/d agonist (GW) and antagonist (GSK) for additional 24 h (^*^*p* < 0.05, ^**^*p* = 0.01–0.001, ^***^*p* < 0.001; One-Way ANOVA-Bonferroni test). GW, GW0742; GSK, GSK0660; DM, DMSO.
