# Supplementary figures and images for: PPARβ/δ and PPARγ maintain undifferentiated phenotypes of mouse adult neural precursor cells from the subventricular zone
Source: Front Cell Neurosci. 2015 Mar 18;9:78. doi: 10.3389/fncel.2015.00078 (PMC4364249; doi:10.3389/fncel.2015.00078)

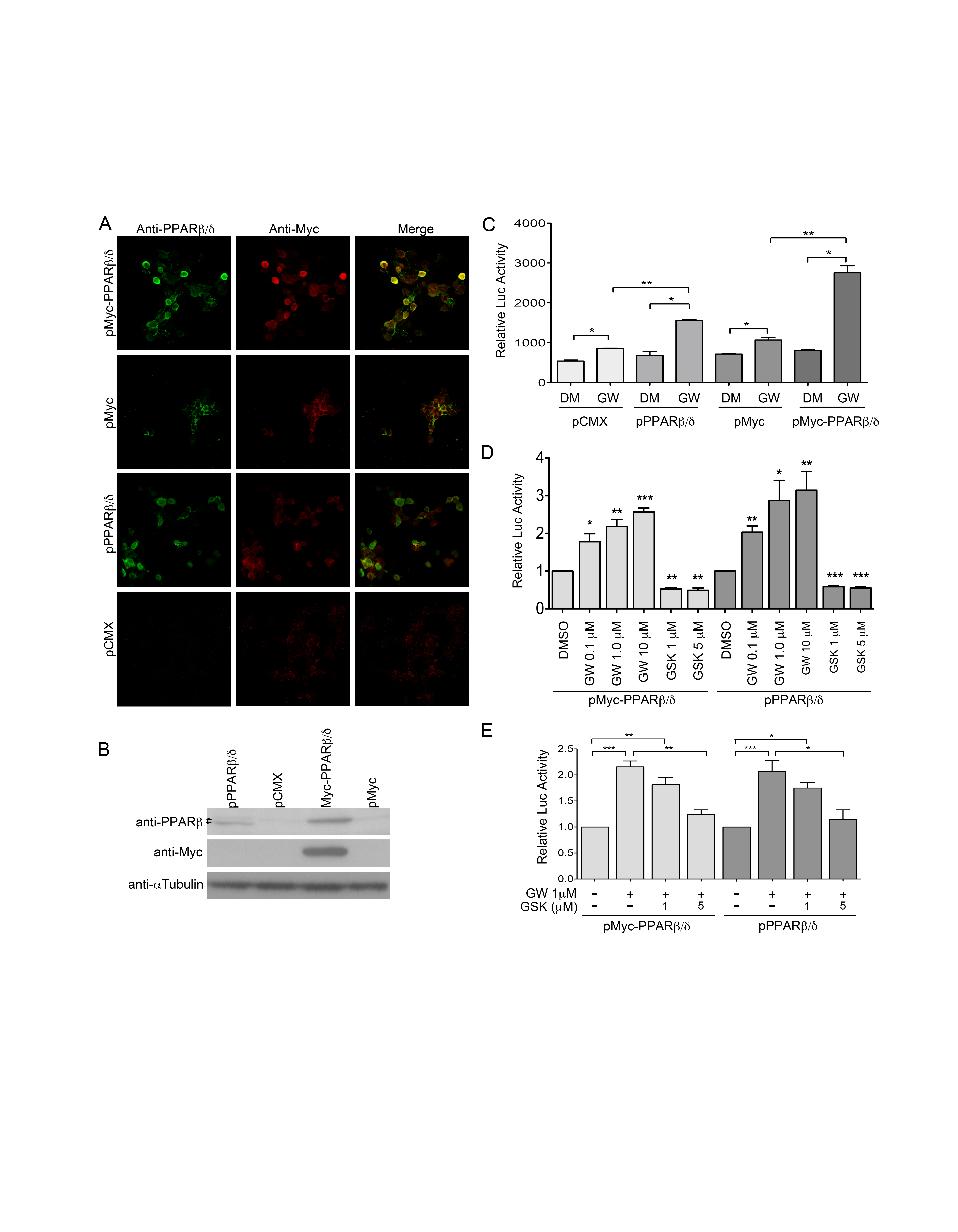

Supplement: Supplementary Figure 1 — pMyc-PPARβ. (A) Double anti-Myc/anti-PPARβ immunofluorescence. Representative confocal microscopy images are shown. (B) Myc-PPARβ expression was evaluated by western blot with anti-PPARβ and anti-Myc antibodies. pPPARβ was used as a control of wild type protein overexpression. pCDNA3-Myc and pCMX are empty vectors for pMyc-PPARβ and pPPARβ, respectively. (C–E) Functional analysis of pMyc-PPARβ by luciferase reporter assay. Cells were transfected with the vectors as indicated plus PPRE luciferase reporter vector and pCMXβ as a control. Luciferase activity was measured and shown in respect to control vector (pCMVβ). (C) Cells were treated with PPARβ/d agonist (GW, 1 μM) or vehicle (DMSO) for 24 h. (D) Different concentrations of GW0742 were used to evaluate activity and to compare both vectors. (E) Cells were pre-treated with antagonist (GSK) for 1 h and then co-treated with PPARβ/d agonist (GW) and antagonist (GSK) for additional 24 h (*p < 0.05, **p = 0.01–0.001, ***p < 0.001; One-Way ANOVA-Bonferroni test). GW, GW0742; GSK, GSK0660; DM, DMSO. [file Image1.TIF]
